# Sulforaphane inhibits proliferation and invasive activity of everolimus-resistant kidney cancer cells *in vitro*

**DOI:** 10.18632/oncotarget.13421

**Published:** 2016-11-17

**Authors:** Eva Juengel, Sebastian Maxeiner, Jochen Rutz, Saira Justin, Frederik Roos, Wael Khoder, Igor Tsaur, Karen Nelson, Wolf O. Bechstein, Axel Haferkamp, Roman A. Blaheta

**Affiliations:** ^1^ Department of Urology, Goethe-University, Frankfurt am Main, Germany; ^2^ Current address: Department of Urology and Pediatric Urology, University Medical Center Mainz, Mainz, Germany; ^3^ Department of Vascular and Endovascular Surgery, Goethe-University, Frankfurt am Main, Germany; ^4^ Department of General and Visceral Surgery, Goethe-University, Frankfurt am Main, Germany

**Keywords:** renal cell carcinoma, complementary and alternative medicine, sulforaphane, proliferation, invasion

## Abstract

Although the mechanistic target of rapamycin (mTOR) inhibitor, everolimus, has improved the outcome of patients with renal cell carcinoma (RCC), improvement is temporary due to the development of drug resistance. Since many patients encountering resistance turn to alternative/complementary treatment options, an investigation was initiated to evaluate whether the natural compound, sulforaphane (SFN), influences growth and invasive activity of everolimus-resistant (RCC^res^) compared to everolimus-sensitive (RCC^par^) RCC cell lines *in vitro*. RCC cells were exposed to different concentrations of SFN and cell growth, cell proliferation, apoptosis, cell cycle, cell cycle regulating proteins, the mTOR-akt signaling axis, adhesion to human vascular endothelium and immobilized collagen, chemotactic activity, and influence on surface integrin receptor expression were investigated. SFN caused a significant reduction in both RCC^res^ and RCC^par^ cell growth and proliferation, which correlated with an elevation in G2/M- and S-phase cells. SFN induced a marked decrease in the cell cycle activating proteins cdk1 and cyclin B and siRNA knock-down of cdk1 and cyclin B resulted in significantly diminished RCC cell growth. SFN also modulated adhesion and chemotaxis, which was associated with reduced expression of the integrin subtypes α5, α6, and β4. Distinct differences were seen in RCC^res^ adhesion and chemotaxis (diminished by SFN) and RCC^par^ adhesion (enhanced by SFN) and chemotaxis (not influenced by SFN). Functional blocking of integrin subtypes demonstrated divergent action on RCC binding and invasion, depending on RCC cell sensitivity to everolimus. Therefore, SFN administration could hold potential for treating RCC patients with established resistance towards everolimus.

## INTRODUCTION

Renal cell carcinoma (RCC) is the most common kidney tumor with more than 330,000 diagnosed cases and more than 140,000 patients succumbing to it every year [1]. Approximately one third of these patients have metastases at diagnosis, and 30–70% of patients with localized disease relapse within 5 years of surgery [2].

During the last decade several targeted drugs have been developed and approved as standard care for patients with metastasized RCC. Licensed target agents include the tyrosine kinase inhibitors, sunitinib and sorafenib, and the mechanistic target of rapamycin (mTOR) inhibitors, temsirolimus and everolimus. Compared to cytokine therapy with interferon a or interleukin-2 [3], these target agents have substantially improved patient outcome, but are not curative since resistance inevitably develops during tyrosine kinase or mTOR inhibitor therapy.

Dissatisfaction, along with strong side effects caused by conventional treatment have driven cancer patients to seek “alternative” and/or “complementary” care options to conventional treatment. “Complementary” is regarded as a non-mainstream approach used in addition to conventional medicine, whereas “alternative” refers to a non-mainstream approach instead of conventional medicine [4]. Complementary and alternative medicine (CAM) has become popular among tumor patients with more than 40% utilizing it worldwide [5]. In Europe, CAM application ranges from 15 to more than 70%, whereby herbal medicine is mostly employed [6]. In recent years, isothiocyanate sulforaphane (SFN), found in cruciferous vegetables such as broccoli and cabbage, has received increasing attention. SFN has been shown to inhibit tumor development and progression by modulating cancer related cell signaling and gene transcription [7]. Inhibition is accomplished by activating apoptosis, by cell cycle arrest, and by preventing metastatic processes [8]. Several epidemiologic studies have revealed a correlation between high intake of SFN rich vegetables and reduced cancer risk [9]. A clinical trial has provided evidence that SFN down-regulates the prostate-specific antigen (PSA) level in men with prostate cancer and decreases biochemical recurrence after radical prostatectomy [10]. Since so many RCC patients turn to CAM once resistance towards conventional therapy occurs, the present study was directed towards investigating whether SFN might indeed hold potential for patients with acquired drug resistance. Accordingly, three RCC cell lines were driven to non-responsiveness towards the mTOR-inhibitor everolimus and the influence of SFN on tumor growth, proliferation, and motility of everolimus-resistant and everolimus-sensitive tumor cells was compared.

## RESULTS

### Growth of everolimus-sensitive and -resistant RCC cells after everolimus application

**Figure 1:**
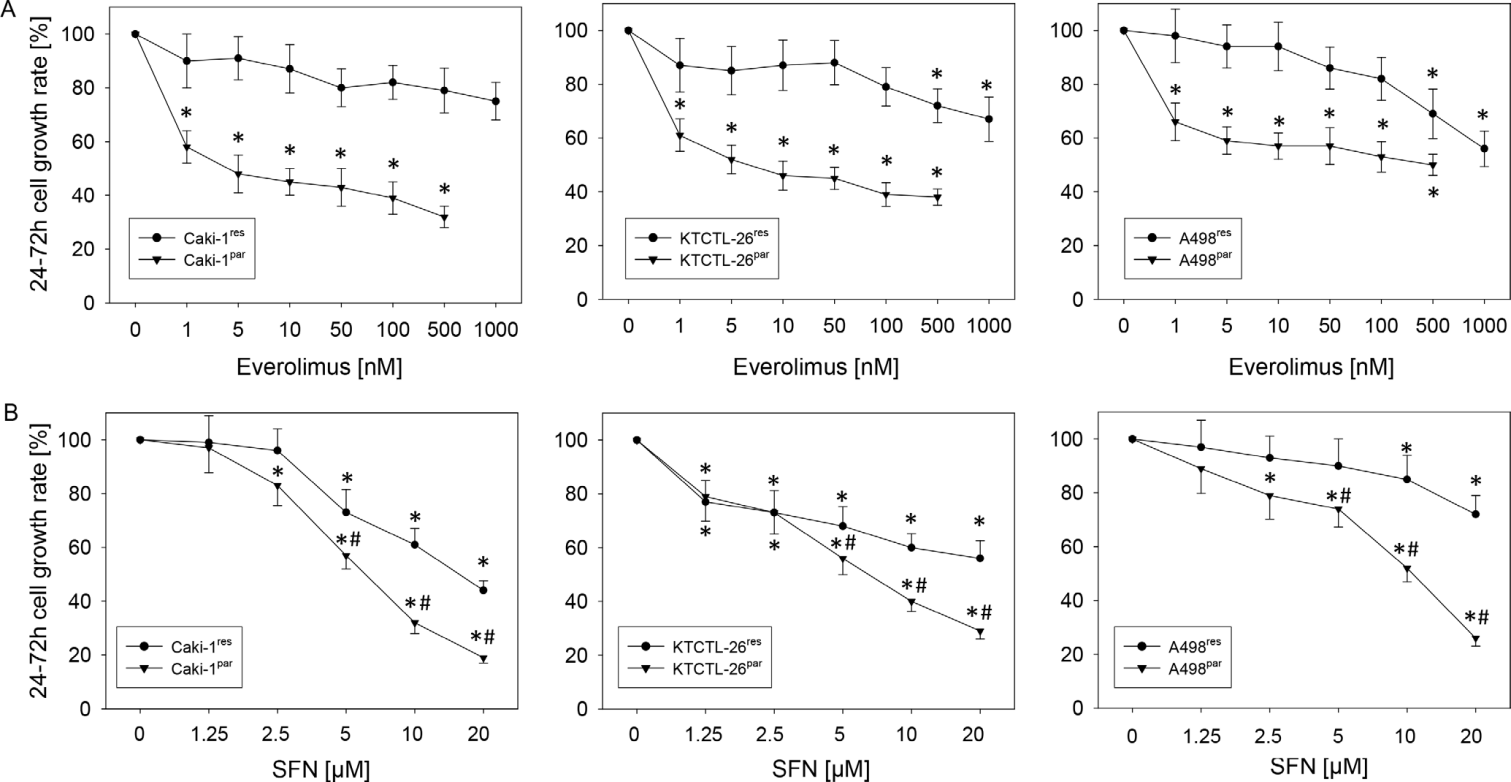
Cell growth (number) in three everolimus sensitive (^par^) and resistant (^res^) RCC cell lines (**A**) everolimus dose dependency. (**B**) SFN dose dependency. Growth was compared to untreated controls, set to 100%. Experiments were done in triplicate and repeated 5 times. * indicates significant difference to controls, ^#^indicates significant difference between everolimus-resistant and -sensitive cells.

Everolimus application caused a dose dependent significant reduction in the number of everolimus-sensitive RCC cells generated over 48 h in all three cell lines, Caki-1^par^, KTCTL-26^par^, and A498^par^ cells. The reduction was already apparent at 1 nM everolimus (Figure 1A). Chronic, previous everolimus exposure induced resistance to acute everolimus application. An everolimus induced reduction in the growth rate greater than 20% was only achieved in KTCTL-26^res^ and A498^res^ cells when they were exposed to an acute dose of 500 or 1000 nM everolimus.

### Growth of everolimus-sensitive and -resistant RCC cells after SFN application

SFN dose-dependently suppressed growth of all three everolimus-sensitive cell lines. It also diminished growth of RCC cells with everolimus-resistance, although the efficacy of SFN was lower in the everolimus-resistant cells, compared to that of the everolimus-sensitive cells (Figure 1B). SFN did not induce early or late apoptosis, as evidenced by the Annexin V-FITC Apoptosis Detection kit.

### Cell cycle shifts during SFN and acute everolimus application

**Figure 2:**
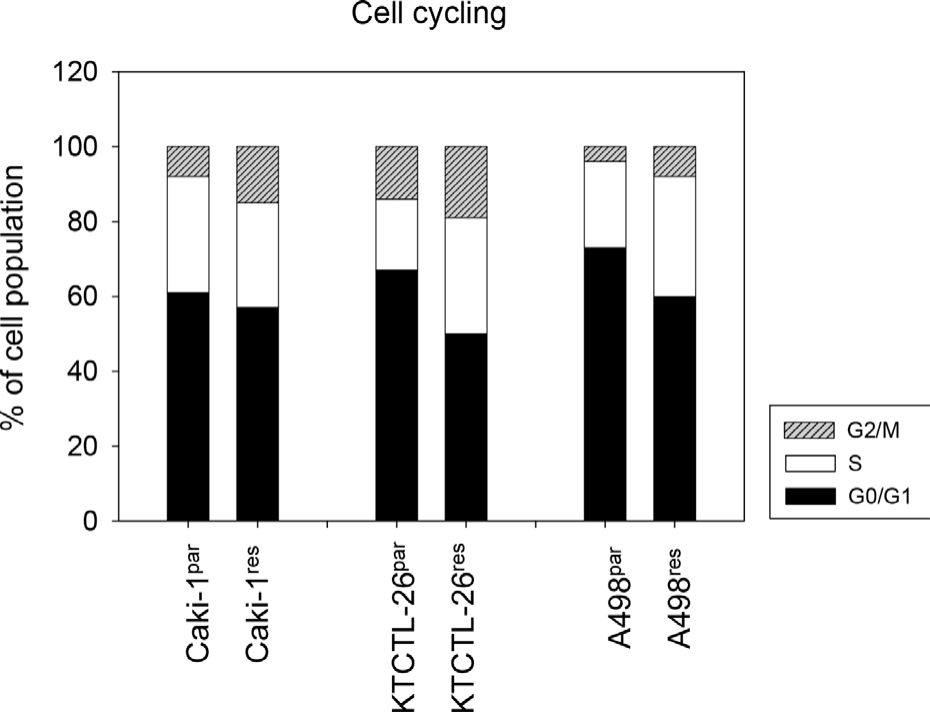
Cell cycle analysis of Caki-1^par^, KTCTL-26^par^ or A498^par^ cells and their everolimus-resistant counterparts (Caki-1^res^, KTCTL-26^res^, A498^res^) The cell population is expressed as percentage of total cells analyzed. One representative experiment of three is shown.

**Figure 3:**
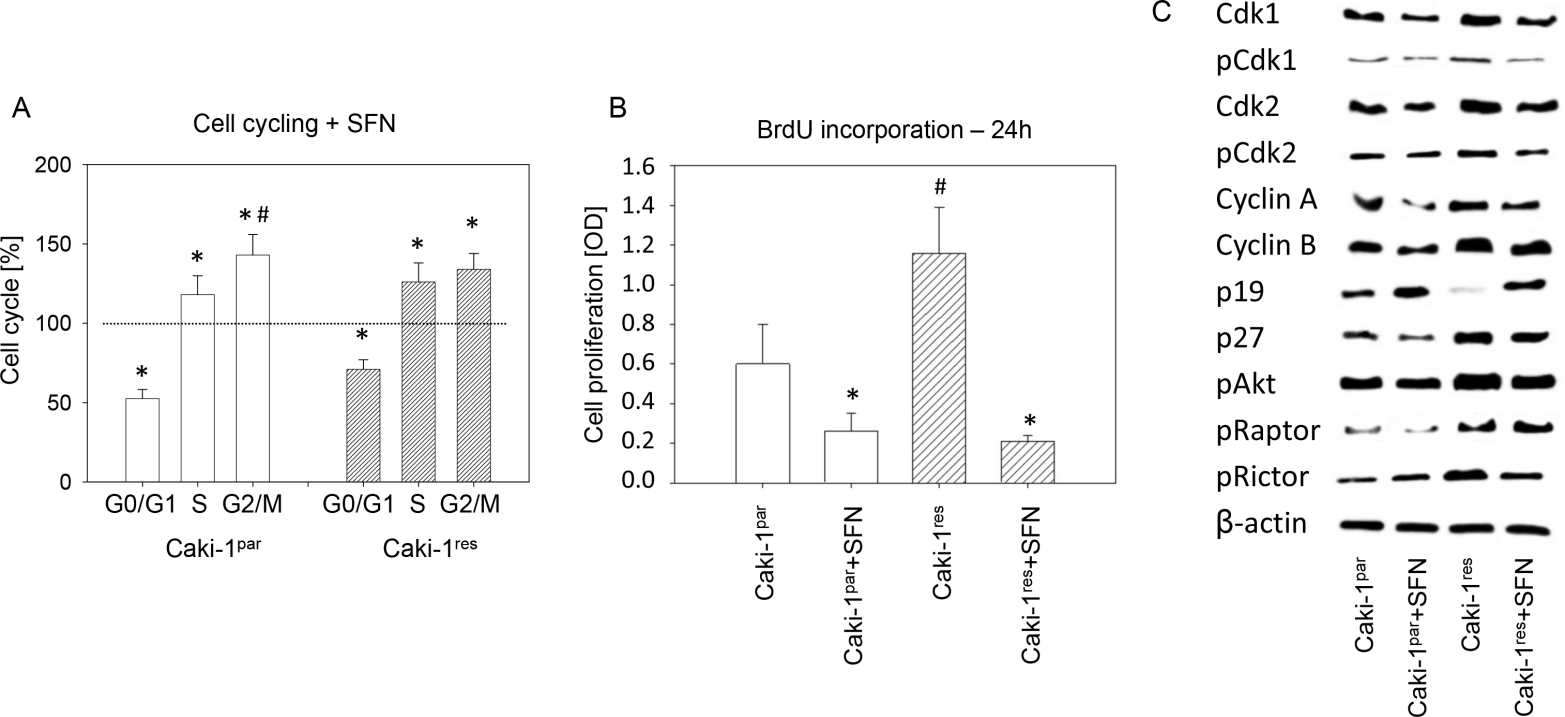
(**A**) Influence of SFN on Caki-1^par^ and Caki-1^res^ cell cycling. SFN (20 μM) was added to the tumor cells and cell cycling evaluated after 24 h. Untreated controls were set to 100%. Experiments were done in triplicate and repeated 5 times. *indicates significant difference to controls. ^#^indicates significant difference between the percentage of S-phase and G2/M-phase cells (**B**) Cell proliferation, evaluated by the BrdU incorporation assay and photometric quantification (OD = optical density). Cells were treated with 20 μM SFN for 24 h. *indicates significant difference to controls not treated with SFN. ^#^indicates significant difference between everolimus-resistant and -sensitive cells. (**C**) Western blot analysis of cell cycle and mTOR related proteins in Caki-1^par^ and Caki-1^res^ cells (SFN treated versus non-treated). β-actin served as internal control. The figure shows one representative of three separate experiments.

Cell cycle analysis revealed an increased percentage of Caki-1^res^ cells in the G2/M-phase and an increased number of KTCTL-26^res^ and A498^res^ cells in the G2/M- and S-phases, compared to respective everolimus-sensitive cells (Figure 2). In both Caki-1^par^ and Caki-1^res^ cells more G2/M- and S-phase cells and less G0/G1-phase cells were apparent when tumor cells were treated with 20 μM SFN for 24 h (Figure 3A). Proliferation was elevated in Caki-1^res^ cells compared to Caki-1^par^ cells, as evidenced by BrdU incorporation (Figure 3B). SFN significantly lowered proliferation of both Caki-1^par^ and Caki-1^res^ cells, as demonstrated by reduced BrdU incorporation (compared to untreated controls).

### SFN alters expression of cell cycle regulating proteins

The influence of SFN on Caki-1^res^ differed from that on Caki-1^par^ in as much as total and activated Cdk1 and Cdk2 were elevated in the everolimus-resistant tumor cells. This was also true with respect to cyclin B, p27, pAkt, pRaptor, and pRictor (Figure 3C). In contrast, p19 was diminished in Caki-1^res^ compared to Caki-1^par^. SFN led to down-regulation of Cdk1, pCdk2, and Cdk2 in both Caki-1^par^ and Caki-1^res^. Cyclin A, cyclin B (cyclin B > cyclin A) and p27 were lowered as well. p19 was up-regulated by SFN. pAkt and pRictor were down-regulated by SFN in Caki-1^res^, whereas pRaptor was down-regulated by SFN in Caki-1^par^.

### Cyclin B-cdk1 knock-down

**Figure 4:**
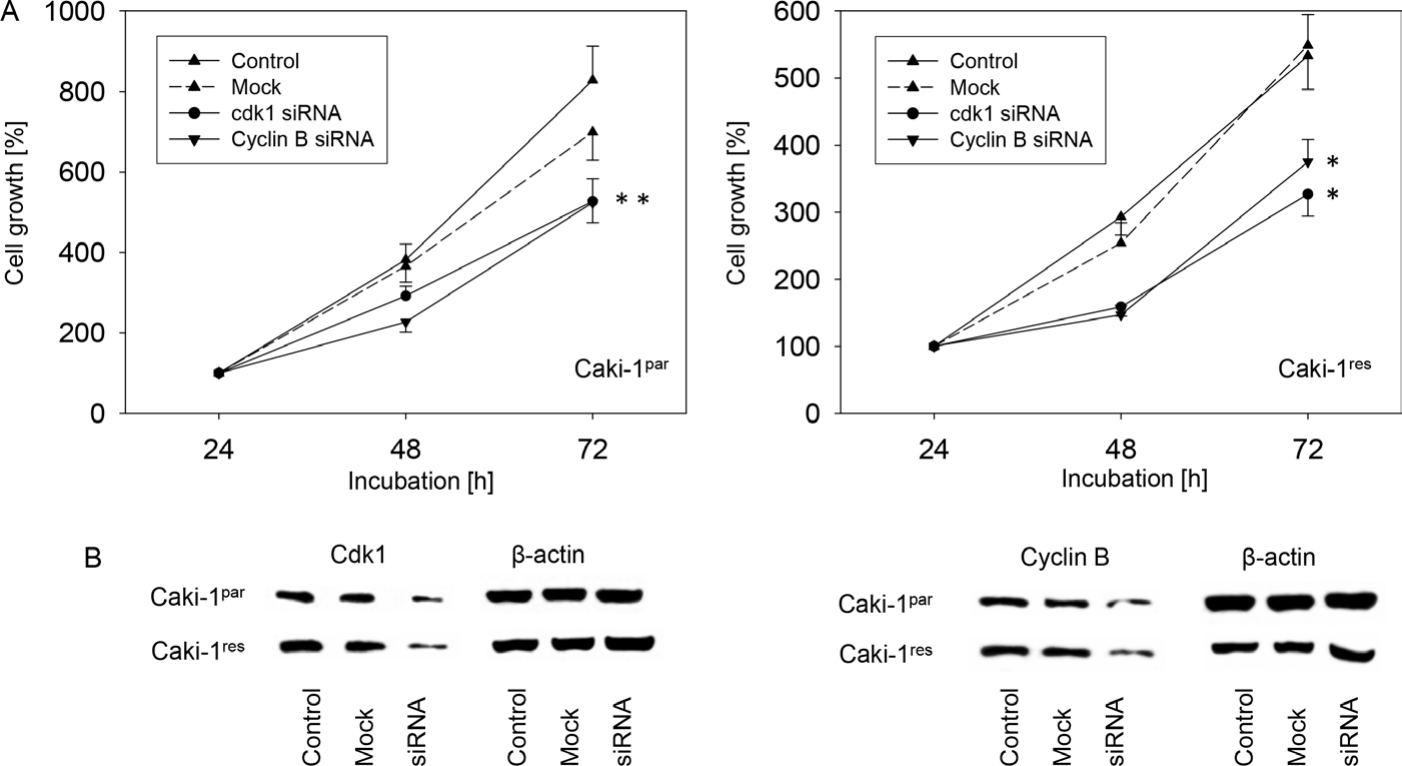
(**A**) Influence of cdk1 or cyclin B knock-down on growth of Caki-1^par^ and Caki-1^res^ cells (24 h value set to 100%). One representative of 6 experiments. *indicates significant difference to controls. (**B**) Western blots show extent of knock-down.

Since cyclin B and cdk1 were strongly down-regulated by SFN in both Caki-1^par^ and Caki-1^res^ cells, this down-regulation could be responsible for the SFN evoked RCC cell growth reduction observed with the MTT assay. Figure 4 shows that treating Caki-1^par^ and Caki-1^res^ with cyclin B or cdk1 specific siRNA reduced the cdk1 and cyclin B content in both cell types. (Figure 4B) This was associated with significant suppression of tumor cell growth (Figure 4A).

### Influence of SFN on tumor cell adhesion and motility

**Figure 5:**
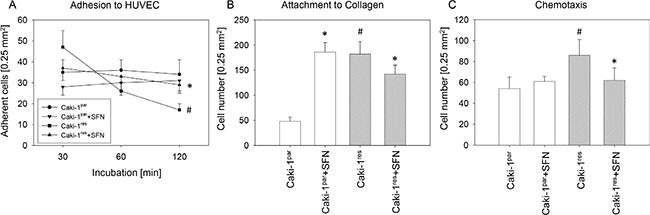
Influence of SFN on adhesion and chemotaxis of everolimus-resistant and everolimus-sensitive Caki-1 cells (**A**) Caki-1^par^ and Caki-1^res^ cell adhesion to HUVEC. (**B**) Adhesion of Caki-1^par^ and Caki-1^res^ cells to immobilized collagen (60 min incubation). (**C**) Chemotactic movement assessed in a Transwell chamber assay with cells seeded in serum-free medium in the upper chamber with 10% FCS as chemoattractant in the lower chamber. A to C show means calculated from five counts. Each diagram represents one of six experiments. *indicates significant difference to controls not treated with SFN. ^#^indicates significant difference between everolimus-resistant and -sensitive cells.

Caki-1^par^ cells rapidly attached to HUVEC with no difference in adherence after 30 and 120 min (Figure 5A). More Caki-1^res^ than Caki-1^par^ cells attached to HUVEC in the initial phase of HUVEC-tumor cell interaction (30 min) but subsequently lost binding capacity, which was lowest after 120 min. SFN significantly down-regulated Caki-1^par^ cell binding to HUVEC (30 and 60 min values, compared to the untreated control). Compared to untreated Caki-1^res^ controls, SFN reduced the number of HUVEC bound Caki-1^res^ cells after 30 min but binding was enhanced after 60 and 120 min (Figure 5A). More Caki-1^res^ than Caki-1^par^ cells adhered to collagen (Figure 5B) and more Caki-1^res^ than Caki-1^par^ crawled underneath the Transwell membrane (Figure 5C). SFN increased the number of Caki-1^par^ but decreased the amount of Caki-1^res^, which attached to immobilized collagen (compared to untreated controls; Figure 5B). SFN also significantly diminished chemotaxis in Caki-1^res^ but not in Caki-1^par^ cells (Figure 5C).

### Influence of SFN on integrin α and β expression in Caki-1^res^ and Caki-1^par^ cells

**Figure 6:**
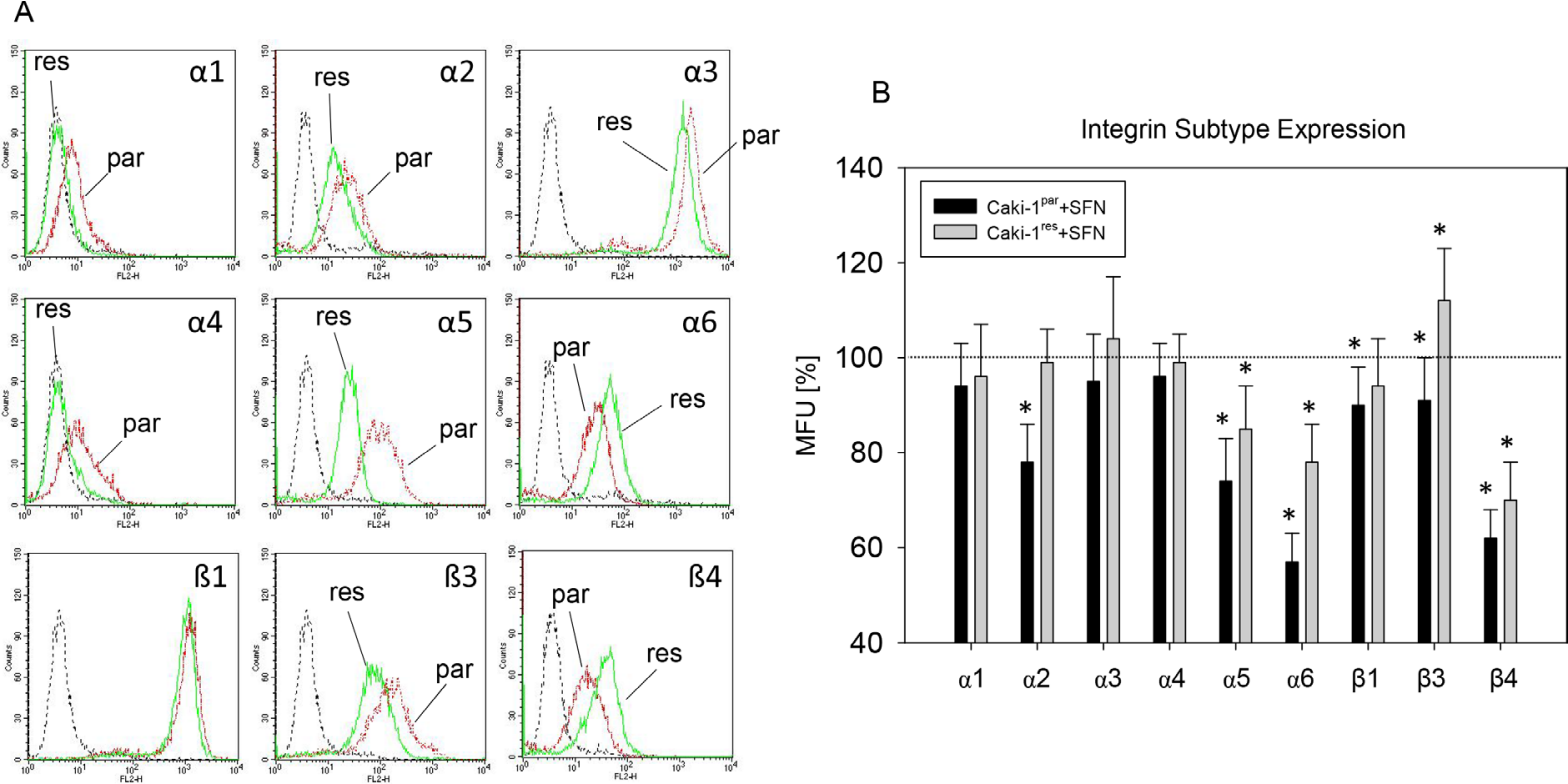
Integrin α and β subtype expression (FACS analysis) on everolimus-resistant (res) and parental (par) Caki-1 cells (**A**) integrin expression; (background fluorescence – dashed lines), Caki-1^par^ and Caki-1^res^ (specific fluorescence - dotted lines). (**B**) integrin subtype expression after 24 h SFN exposure (20 μM), compared to untreated controls set to 100%. *indicates significant difference to controls. MFU: mean fluorescence units.

Caki-1^par^ revealed a slight or moderate expression of the integrin subtypes α1, α2, α4, α6, and β4. Strong expression was recorded for the integrins α3, α5, β1, and β3 (Figure 6A). Everolimus-resistance was associated with diminished expression of the integrins α1 – α5 and β3 (compared to everolimus-sensitive controls). β1 integrin expression was the same in everolimus-resistant and -sensitive cells, but integrins α6 and β4 were enhanced in everolimus-resistant cells. SFN caused a reduction in integrins α5, α6, β1, and β4 with stronger effects seen in Caki-1^par^ than in Caki-1^res^. SFN caused integrin β3 to become down-regulated in Caki-1^par^ but up-regulated in Caki-1^res^, whereas integrin α2 was down-regulated in Caki-1^par^ but remained unchanged in Caki-1^res^ cells (Figure 6B).

### Integrin α5, α6, and β4 blockade differentially influence Caki-1^res^ and Caki-1^par^ adhesion and migration

**Figure 7:**
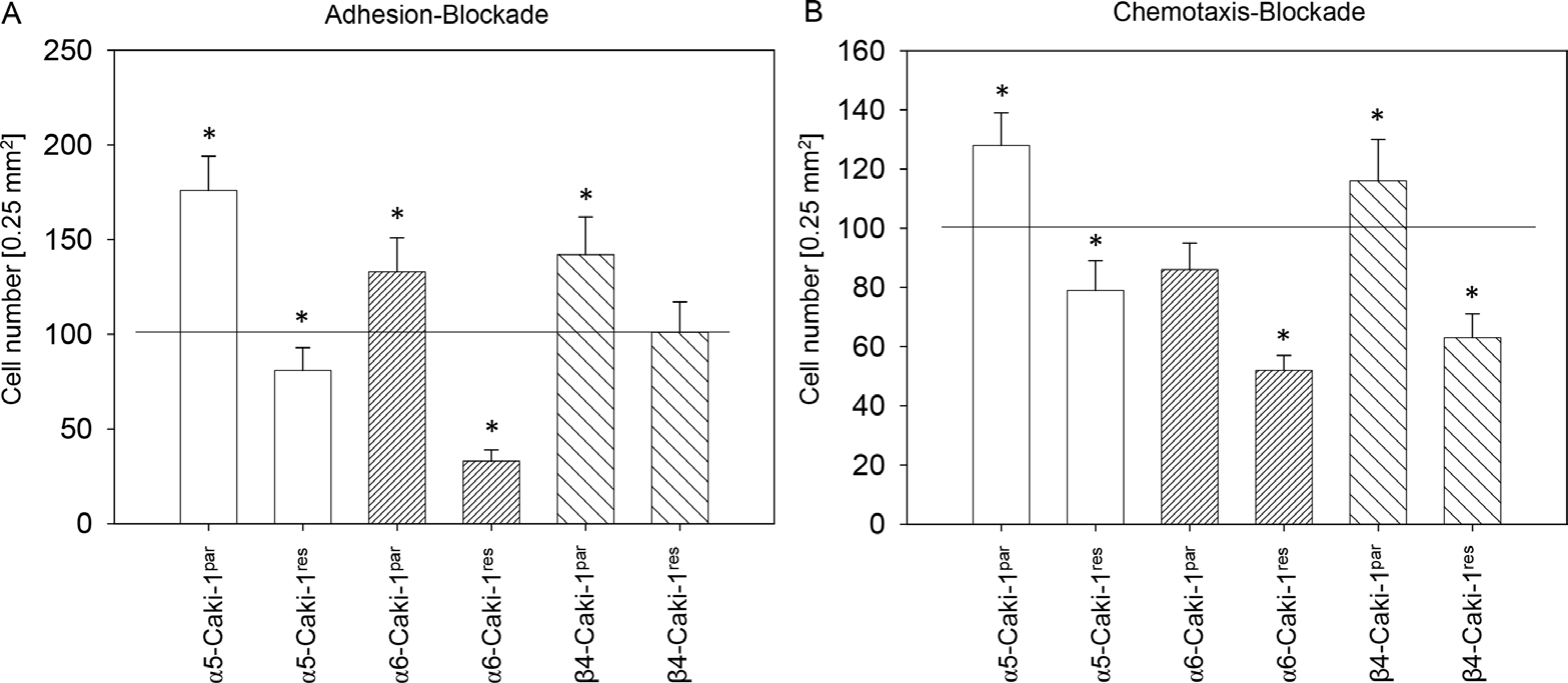
Influence of integrin α5, α6, or β4 blockade on Caki-1^par^ and Caki-1^res^ cell adhesion to immobilized collagen (**A**) and on chemotaxis (**B**). Cells were preincubated for 60 minutes with a function-blocking anti–integrin mAb. Controls were untreated and set to 100%. *indicates significant difference to controls.

Since SFN strongly altered integrin α5, α6, and β4 expression, the relevance of these integrin types for the adhesive and motile behaviour of the everolimus-sensitive and everolimus-resistant Caki-1 cells was investigated. Integrin blockade differentially influenced adhesion and chemotaxis of Caki-1^par^ compared to Caki-1^res^ cells (Figure 7). Blocking α5 triggered enhanced adhesion (Figure 7A) and chemotactic (Figure 7B) behavior of Caki-1^par^ , but diminished adhesion and chemotactic behavior in Caki-1^res^ cells. A similar phenomenon was observed when Caki-1 cells were treated with α6 or β4 function-blocking antibodies, with the exception that adhesion of Caki-1^res^ cells was not altered by integrin β4 blockade and chemotaxis of Caki-1^par^ was not modulated by integrin α6 blockade.

## DISCUSSION

Resistance to everolimus in RCC cells was associated with increased mitotic activity, loosening of RCC-HUVEC contacts, increased interaction with a collagen matrix and elevated chemotactic movement. Earlier investigations have shown that everolimus-resistance drives RCC cells towards high proliferation and motility [11, 12]. SFN not only reduced growth of everolimus-sensitive tumor cells but also counteracted aggressive proliferative and invasive activity of everolimus-resistant RCC cell lines.

The effect was most prominent with a concentration of 20 μM. Whether this concentration can be attained under therapeutic conditions in tumor patients remains to be seen, since no pertinent studies are available. Cipolla et al., in treating prostate cancer patients with SFN, employed an oral dose of 60 mg/day, but provided no further details about SFN metabolism or bioavailability [10]. The urine concentration of dithiocarbamates, a group of SFN-metabolites, has been evaluated in clinical trials with healthy volunteers to be about 20 μM after consuming 50 g/day [13] or 70 g/day broccoli sprouts [14].

In the presence of SFN cell cycle analysis revealed tumor cell accumulation in the S- and G2/M-phase, with accumulation in the G2/M-phase being greater than in the S-phase in the parental (everolimus-sensitive) sublines. SFN induced G2/M phase-arrest has also been observed in osteosarcoma [15], bladder [16], colon [17], ovarian [18], prostate [19], and breast cancer cells [20]. This G2/M phase-arrest corroborates the SFN induced decrease in RCC proliferation found in the present investigation. An SFN induced down-regulation of the cyclin B-cdk1 axis also occurred in both everolimus-sensitive and -resistant RCC. The G2/M phase arrest, coupled with down-regulation of the cyclin B-cdk1 axis, caused by SFN indicates that SFN inhibits mitotic processes.

Though SFN has been shown by other investigators to reduce cdk1 [21, 22], it is not the rule. An increase in cyclin B has been documented in bladder and prostate cancer cells [23, 24] but no change has been reported in colon cancer-derived tumors [25], indicating that the influence of SFN may depend on the tumor entity. The action of SFN on cyclin A also demonstrates dependence on the tumor entity. In the present investigation cyclin A was down-regulated in RCC cells. It has also been reported to be down-regulated in osteosarcoma [26] and colon carcinoma cells [27]. However, down-regulation has not been reported in oral carcinoma cells [28].

The cell cycle regulator, p19, was up-regulated in both Caki-1^par^ and Caki-1^res^ by SFN. This observation is corroborated by several other investigations demonstrating a negative association between p19 expression and proliferative activity in a panel of solid tumor types [29–31]. It, therefore, seems likely that SFN triggered elevation of p19, along with cdk-cyclin down-regulation, contributes to the inhibition of RCC cell growth and proliferation.

Since p27 serves as a tumor suppressor, it was expected that SFN would induce enhanced expression. However, this expectation was foiled since application of SFN caused diminished p27 expression in both everolimus-sensitive and -resistant tumor cells. Other investigators have reported that applying SFN to several tumor cell lines (but not to RCC cells) leads to p27 induction [32, 33]. Another investigator found no modification of p27 by SFN [34]. Mans et al. concluded that p27 induces tumor cell senescence in RCC patients [35]. This is in line with other investigators showing loss of p27 to correlate with RCC recurrence and cancer-related patient death [36]. Meanwhile, newer interpretation has been advanced associating high cytoplasmic p27 expression with worse cancer-specific survival in RCC [37]. Presumably, shifting p27 from the nuclear to the cytoplasmic compartment could be predictive for poor outcome in RCC [38]. A further aspect should also be considered. Besides cell growth regulation, p27 also modifies cell-cell interaction, driving RCC cell invasion and metastatic progression forward [39]. Speculatively, SFN induced loss of p27 might hinder RCC cells in crossing the endothelial cell barrier and settling as secondary tumors. The alteration of adhesive and invasive behavior of RCC cells by SFN seen in our *in vitro* assays, at least partially, corroborates the assumption that diminished p27 expression not only contributes to cell growth regulation but also prevents RCC migration.

SFN exerted different influences on Caki-1^par^ and Caki-1^res^. The total tumor cell number was reduced more when SFN was applied to everolimus-sensitive RCC cells, compared to everolimus-resistant cells. Sensitive tumor cells were mainly driven into the G2/M phase, whereas the resistant cells were equally driven into the G2/M and S-phases. The SFN induced inhibition of proliferation was more intense in everolimus-resistant than in -sensitive cells. The differences in tumor cell biology were accompanied by differences in cell cycle protein modifications. SFN caused diminished pCdk1 expression in everolimus-resistant but not in -sensitive RCC cells, whereas Cyclin A was reduced in the everolimus-sensitive but not in the -resistant RCC cells. SFN suppressed pRaptor in the everolimus-sensitive and pRictor in the everolimus-resistant RCC cells. Since resistance is characterized by altered cell signaling machinery, it is not surprising that molecules within the signaling cascade are altered differently in Caki-1^res^ and Caki-1^par^ cells when SFN is applied. With respect to the mTOR sub-members Rictor and Raptor, these protein complexes individually modify cell cycle progression and proliferation [40, 41]. Divergent regulation of pRictor and pRaptor, depending on everolimus sensitivity, might therefore account for the different influence of SFN on the Caki-1 cell lines.

Different responses of Caki-1^res^ and Caki-1^par^ cells to SFN were also apparent with respect to adhesion and chemotaxis. Only a slight reduction in Caki-1^par^ cells bound to HUVEC was induced by SFN, attachment to collagen was enhanced and motile behavior was not influenced at all. In strong contrast, SFN prevented Caki-1^res^ from becoming highly adhesive or highly motile. More Caki-1^res^ cells attached to HUVEC after 120 min incubation in the presence of SFN, fewer cells bound to collagen and only a few cells migrated. These effects on the everolimus-resistant tumor cells open the possibility that SFN might be a treatment option once tumors have become non-responsive to conventional drug treatment. SFN has recently been shown to reduce the metastatic potential of drug-resistant breast cancer cells *in vitro* [42]. There is also evidence that SFN might overcome chemoresistance towards adriamycin, cisplatin [43], doxorubicin [44], and paclitaxel [45]. The current results demonstrate high efficacy of SFN in reducing the metastatic potential of everolimus-resistant RCC cells *in vitro*. Whether SFN can re-establish everolimus sensitivity is currently under investigation.

SFN caused down-regulation of the integrin adhesion receptors α5, α6, and β4 subtypes in both Caki-1^par^ and Caki-1^res^ cells. This consistent down-regulation was accompanied by enhanced binding of Caki-1^par^ cells, but blocked binding and chemotaxis of Caki-1^res^, indicating different integrin function, depending on the drug responsiveness of the tumor cells. For example blocking of α5 was accompanied by elevated adhesion and chemotaxis of Caki-1^par^, but by reduced adhesion and chemotaxis of Caki-1^res^ cells. Loss of β4 integrin enhanced motile activity of Caki-1^par^, but reduced that of Caki-1^res^ cells. Blockade of integrin α6 led to up-regulation of Caki-1^par^ adhesion, but to a down-regulation of Caki-1^res^ adhesion. Divergent activity of a particular integrin is not uncommon and we have recently reported that integrins undergo functional changes during the process of resistance development, driving tumor cells to become highly invasive [11, 46]. This disparity in integrin function may explain why SFN treatment caused enhanced binding of everolimus-sensitive RCC cells to collagen but reduced binding in everolimus-resistant cells. Diminished α5, α6, and β4 expression after SFN application could be responsible for the diminished chemotactic behavior of Caki-1^res^. Chemotaxis of Caki-1^par^ cells was not influenced by SFN. Possibly, integrin α6 reduction is irrelevant to motile spreading of everolimus-sensitive cells and/or because integrin α2 suppression, induced by SFN exclusively in Caki-1^par^, might correlate with decreased chemotaxis [47], (thereby counteracting the pro-chemotaxic effects induced by α5 and β4 reduction). However SFN actually functions, it triggers firm attachment of everolimus–sensitive RCC to the collagen matrix, thereby indirectly preventing invasive progression. In contrast, everolimus-resistant cells are modified by SFN through direct inhibition of both adhesion and chemotaxis. The direct action of SFN on adhesion and penetration could be of potential importance in treating patients with everolimus-resistant cancer.

Overall, SFN has been demonstrated to act on a panel of RCC cell lines by reducing tumor growth and proliferation *in vitro*. Verification in animals with acquired everolimus resistant cancer must reveal whether the potential of SFN seen *in vitro* can be substantiated *in vivo*. If verified, then SFN might be of value in supporting conventional therapy with an mTOR inhibitor in patients with acquired resistance.

## MATERIALS AND METHODS

### Cells and cell culture

A panel of three RCC cell lines were initially investigated (Caki-1, KTCTL-26, and A498). The effects of everolimus and L-sulforaphane on cell growth and the cell cycle were tested on all three of these cell lines in an everolimus-sensitive and an everolimus-resistant state. Furthergoing investigation including cell proliferation, apoptosis, cell cycle regulating proteins, the mTOR-akt signaling axis, adhesion to human vascular endothelium and immobilized collagen, chemotactic activity, and influence on surface α and β integrin receptor expression were carried out only on Caki-1 cells.

Caki-1 and KTCTL-26 cells were purchased from LGC Promochem (Wesel, Germany) and A498 cells from Cell Lines Service (Heidelberg, Germany). Tumor cells were grown and subcultured in RPMI 1640 medium (Seromed, Berlin, Germany) supplemented with 10% fetal calf serum (FCS), 20 mM HEPES-buffer, 1% glutamax and 1% penicillin/streptomycin (all: Gibco/Invitrogen; Karlsruhe, Germany) at 37°C in a humidified, 5% CO_2_ incubator. Subcultures from passages 5–24 were employed.

Human umbilical vein endothelial cells (HUVEC) were harvested by enzymatic treatment with dispase (Gibco/Invitrogen) and cultured in Medium 199 (M199; Biozol, Munich, Germany), supplemented with 10% FCS, 10% pooled human serum, 20 μg/ml endothelial cell growth factor (Boehringer, Mannheim, Germany), 0.1% heparin, 100 ng/ml gentamycin and 20 mM HEPES-buffer (pH 7.4). Subcultures from passages 2–6 were employed.

### Drug treatment

Everolimus (Novartis Pharma AG, Basel, Switzerland) was dissolved in DMSO as a 10 mM stock solution and stored in aliquots at −20°C. Prior to experiments, everolimus was diluted in cell culture medium. Cell growth experiments were carried out in the presence of 1 – 1000 nM everolimus. Resistance towards everolimus was induced by subjecting Caki-1, KTCTL-26, or A498 cells with stepwise ascending concentrations from 1 nM up to 1 μM. The tumor cells were further exposed to 1 μM everolimus twice weekly for over a year. Tumor cells, resistant to everolimus, were designated Caki-1^res^, KTCTL-26^res^, and A498^res^. Control cells, sensitive to everolimus, were designated Caki-1^par^, KTCTL-26^par^, and A498^par^. L-Sulforaphane was provided by Biomol, Hamburg, Germany. Concentrations from 1.25 – 20 μM SFN were applied to cell cultures to evaluate effects on the growth of both everolimus- resistant and everolimus-sensitive tumor cells. Since 20 μM SFN showed the greatest growth inhibitory effect, all further experiments were carried out with 20 μM SFN. Controls remained untreated. To evaluate toxic effects of everolimus and SFN, cell viability was determined by trypan blue (Gibco/Invitrogen, Darmstadt, Germany).

### Apoptosis

To detect apoptosis the expression of Annexin V/propidium iodide (PI) was evaluated using the Annexin V-FITC Apoptosis Detection kit (BD Pharmingen, Heidelberg, Germany). Tumor cells were washed twice with PBS-buffer and then incubated with 5 μl of Annexin V-FITC and 5 μl of PI in the dark for 15 min at room temperature. Cells were analyzed on a FACScalibur (BD Biosciences, Heidelberg, Germany). The percentage of vital, necrotic, and apoptotic cells (early and late) in each quadrant was calculated using Cell-Quest software (BD Biosciences).

### Measurement of tumor cell growth and proliferation

Cell growth was assessed using the 3-(4,5- dimethylthiazol-2-yl)-2,5-diphenyltetrazolium bromide (MTT) dye reduction assay (Roche Diagnostics, Penzberg, Germany). RCC cells (50 μl, 1 × 10^5^ cells/ml) were seeded onto 96-well tissue culture plates. After 24, 48, and 72 h MTT (0.5 mg/ml) was added for an additional 4 h. Subsequently, cells were lysed in a buffer containing 10% SDS in 0.01 M HCl. The plates were incubated overnight at 37°C, 5% CO_2_. Absorbance at 550 nm was determined for each well using a microplate ELISA reader. Each experiment was done in triplicate. After subtracting background absorbance, results were expressed as 24 – 72 h cell growth rate calculated in percentage increase compared to controls set at 100%.

Cell proliferation was measured using a BrdU cell proliferation enzyme-linked immunosorbent assay (ELISA) kit (Calbiochem/Merck Biosciences, Darmstadt, Germany). Tumor cells, seeded onto 96-well microtitre plates, were incubated with 20 μl BrdU-labeling solution per well for 8 h, and then fixed and detected using anti-BrdU mAb according to the manufacturer's instructions. Absorbance was measured at 450 nm.

### Cell cycle analysis

Cell cycle analysis was performed with everolimus-resistant and -sensitive subconfluent RCC cultures. Tumor cell populations were stained with propidium iodide using a Cycle TEST PLUS DNA Reagent Kit (BD Pharmingen, Heidelberg, Germany) and then subjected to flow cytometry with a FACScan flow cytometer (Becton Dickinson). 10,000 events were collected from each sample. Data acquisition was carried out using Cell-Quest software and cell cycle distribution, calculated with ModFit software (BD Biosciences). The number of gated cells in the G1-, S-, or G2/M-phases was expressed as % of total cells.

### Western blot analysis of cell cycle regulating proteins

To investigate cell cycle regulating proteins, tumor cell lysates were applied to a 7% polyacrylamide gel and electrophoresed for 90 min at 100 V. The lysis buffer consisted of Tris-NaCl, 10% Tergitol, 0.25% Na-deoxycholate, 1 mM EDTA, 1 mg/ml aprotinin, 1 mg/ml leupeptin, 1 mg/ml pepstatin, 2 mM NaF, 2 mM Na_3_VO_4_, 2 mM PMSF. Protein was then transferred to nitrocellulose membranes (1 hr, 100 V). After blocking with non-fat dry milk for 1 hr, the membranes were incubated overnight with monoclonal antibodies directed against the following cell cycle proteins (all from BD Biosciences): Cdk1 (IgG1, clone 1), phospho-Cdk1 (pY15; IgG1, clone 44), Cdk2 (IgG2a, clone 55), phospho-Cdk2 (Thr160; MerckMillipore, Darmstadt, Germany), cyclin A (IgG1, clone 25), cyclin B (IgG1, clone 18), p19 (IgG1, clone 52/p19), p27 (IgG1, clone 57; all from BD Biosciences). The following monoclonal antibodies were employed to determine mTOR signaling: phospho-Akt (pAkt; clone 104A282, mouse IgG1, BD Biosciences), phospho-rictor (pRictor; IgG, Thr1135, D30A3), phospho-raptor (IgG, Ser792; both MerckMillipore). HRP-conjugated goat-anti-mouse IgG (Upstate Biotechnology, Lake Placid, NY, USA; dilution 1:5000) served as the secondary antibody. The membranes were briefly incubated with ECL detection reagent (ECL^™^, Amersham/GE Healthcare, München, Germany) to visualize the proteins and then analysed by the Fusion FX7 system (Peqlab, Erlangen, Germany). β-actin (1:1000; Sigma-Aldrich, Taufenkirchen, Germany) served as the internal control.

### RCC cell adhesion

To analyze Caki-1 adhesion, HUVECs were seeded onto six-well multiplates (Falcon Primaria; BD Biosciences, Heidelberg, Germany) in complete HUVEC medium. When confluency was reached, Caki-1 cells (everolimus-resistant and -sensitive, SFN treated and non-treated controls) were detached from their culture flasks by Accutase treatment (PAA Laboratories, Cölbe, Germany). Tumor cells (0.5 × 10^6^) were then added to the HUVEC monolayer for 30, 60, or 120 minutes. Subsequently, non-adherent cells were washed off using warmed (37°C) M199. Adherent cells were fixed with 1% glutaraldehyde and counted in five different fields, each 0.25 mm^2^ , using a phase-contrast microscope. Mean cellular adhesion in the five fields was calculated.

### Attachment to a collagen matrix

Six-well plates (Falcon Primaria) were coated with collagen G [extracted from calfskin, consisting of 90% collagen type I and 10% collagen type III; diluted to 400 μg/ml in phosphate-buffered saline (PBS); Seromed, Berlin, Germany] overnight. Plastic dishes served as the background control. Plates were washed with 1% BSA in PBS to block nonspecific cell adhesion. Caki-1 cells (0.5 × 10^6^) were then added to each well for 60 minutes. Subsequently, non-adherent tumor cells were washed off, and the remaining adherent cells were fixed with 1% glutaraldehyde and counted under a microscope. Mean cellular adhesion, defined by adherent cells_coated well_ − adherent cells_background_, was calculated from five different observation fields (5 × 0.25 mm^2^).

### Tumor cell chemotaxis

Serum-induced chemotactic movement was investigated using six-well Transwell chambers (Greiner Bio-One, Frickenhausen, Germany) with 8-μm pores. Caki-1 cells (0.5 × 10^6^/ml) were placed in the upper chamber in serum-free medium. The lower chamber contained 10% serum. After 20-hour incubation, the upper surface of the Transwell membrane was gently wiped with a cotton swab to remove cells that had not migrated. Cells, which had moved to the lower surface of the membrane, were stained using hematoxylin and counted under a microscope. Mean chemotaxis was calculated from five different observation fields (5 × 0.25 mm^2^).

### Integrin surface expression

Caki-1^res^ and Caki-1^par^ cells were detached from their culture flasks by Accutase and washed in blocking solution (PBS, 0.5% BSA). The cells were then incubated for 60 minutes at 4°C with phycoerythrin (PE)-conjugated monoclonal antibodies (mAbs) directed against the following integrin subtypes: anti-α1 (mouse IgG1, clone SR84), anti-α2 (mouse IgG2a, clone 12 F1-H6), anti-α3 (mouse IgG1, clone C3 II.1), anti-α4 (mouse IgG1, clone 9 F10), anti-α5 (mouse IgG1, clone IIA1), anti-α6 (rat IgG2a, clone GoH3), anti-β1 (mouse IgG1, clone MAR4), anti-β3 (mouse IgG1, clone VI-PL2), or anti-β4 (rat IgG2a; clone 439–9B; all: BD Biosciences). Tumor cell integrin expression was then measured using a FACScan (BD Biosciences; FL-2H (log) channel histogram analysis; 1 × 10^4^ cells per scan) and expressed as mean fluorescence units. A mouse IgG1-PE (MOPC-21) or IgG2a-PE (G155–178; all: BD Biosciences) was used as an isotype control.

### Blocking and knock-down studies

Caki-1 everolimus-resistant and –sensitive (parental) cells were incubated for 60 minutes with 10 μg/ml function-blocking anti–integrin α5 (clone P1D6), α6 (clone NKI-GoH3) or β4 (clone ASC-8; all: MerckMillipore). Control cells were incubated with cell culture medium alone. Adhesion and chemotaxis were then evaluated as previously described. In further experiments, Caki^par^ and Caki^res^ cells (3 × 10^5^/well) were transfected with small interfering RNA (siRNA) directed against cdk1 (gene ID: 983, target sequence: AAGGGGTTCCTAGTACTGCAA; Qiagen, Hilden, Germany) or cyclin B (gene ID: 891, target sequence: AATGTAGTCATGGTAAATCAA; Qiagen), with an siRNA/transfection reagent (HiPerFect Transfection Reagent; Qiagen) ratio of 1:6. Non-treated cells and cells treated with 5 nM control siRNA (All stars negative control siRNA; Qiagen) served as controls. Subsequently, tumor cell growth was analyzed as previously described.

### Statistics

All experiments were performed three to six times. Statistical significance was calculated with the Wilcoxon–Mann-Whitney *U* test. Differences were considered statistically significant at *P* < 0.05.
